# Health related quality of life for young people receiving dialectical behaviour therapy (DBT): a routine outcome-monitoring pilot

**DOI:** 10.1186/s40064-016-2826-9

**Published:** 2016-07-20

**Authors:** M. Swales, R. A. B. Hibbs, L. Bryning, R. P. Hastings

**Affiliations:** North Wales Adolescent Service, Betsi Cadwaladr University Health Board, Abergele, UK; North Wales Clinical Psychology Programme, School of Psychology, Bangor University, Bangor, Wales, UK; Integral Business Support Ltd, Wrexham, UK; Centre for Health Economics and Medicines Evaluations, Bangor University, Bangor, Wales, UK; CEDAR, University of Warwick, Coventry, UK

**Keywords:** Adolescents, Borderline personality disorder, Health-related quality-of-life, Routine outcome monitoring

## Abstract

**Purpose:**

Adults presenting with borderline personality disorder (BPD) score poorly on measures of health related quality of life (HRQoL). Little is known about HRQoL in adolescents with BPD type presentations and how treatment impacts quality of life. Our primary aim was to use routinely collected quality-of-life outcome measures pre and post-treatment in dialectical behaviour therapy (DBT) for adolescents to address this gap. Secondary aims were to benchmark these data against EuroQol 5 dimensions (EQ-5D™) outcomes for clients treated in clinical trials and to assess the potential of the EQ-5D™ as a benchmarking tool.

**Method:**

Four adolescent DBT teams, routinely collecting outcome data using a pseudonymised secure web-based system, supplied data from consecutive discharges.

**Results:**

Young people in the DBT programmes (n = 43) had severely impaired HRQoL scores that were lower at programme admission than those reported in published studies using the EQ-5D™ in adults with a BPD diagnosis and in one study of adolescents treated for depression. 40 % of adolescents treated achieved Reliable Clinical Change. HRQoL improved between admission and discharge with a large effect size. These results were not statistically significant when clustering in programme outcomes was accounted for.

**Conclusion:**

Young people treated in NHS DBT programmes for BPD type presentations had poorer HRQoL than adults with a BPD diagnosis and adolescents with depression treated in published clinical trials. The EQ-5D™ detected reliable change in this group of adolescents. Programme outcome clustering suggests that both the measure and the web-based monitoring system provide a mechanism for benchmarking clinical programmes.

## Background

Whilst borderline personality disorder (BPD) is most commonly diagnosed in adults, more recently clinicians and researchers have begun to consider the assessment and identification of personality disorders in adolescents (Miller et al. 2008; Sharp and Fonagy 2015). Studies investigating BPD traits in young people report that these presentations predict the presence of personality disorders in adulthood and are also linked to other psychiatric disorders, impaired long-term functioning and to increased mortality (Cohen et al. 2005; Crawford et al. 2008; Winograd et al. 2008). Currently, there are no established effective treatments for young people with BPD-type presentations. More typically, adolescent research has focussed on interventions for repeated self-harm, one of the diagnostic criteria for BPD. A meta-analysis of psychological and social interventions for suicide attempts and self-harm (Ougrin et al. 2015) reviewing 19 trials comprising 2176 young people concluded that the selected interventions appeared to be effective overall for self-harm. Dialectical behaviour therapy (DBT) (Miller and Rathus 2002; Mehlum et al. 2014), cognitive-behavioural therapy (CBT) (Esposito-Smythers et al. 2011) and mentalization-based therapy (MBT) (Roussow and Fonagy 2012) demonstrated the largest effect-sizes; however, these results require independent replication.

DBT is an effective treatment for BPD in adults with robust evidence from randomised controlled trials demonstrating significant impacts on a number of important outcomes, including reduced suicidal and self-harming behaviours and service utilisation (Clarkin et al. 2007; Koons et al. 2001; Linehan et al. 1991, 1999, 2002, 2006; McMain et al. 2009; Verheul et al. 2003). The recent Cochrane Review concluded that DBT was the only psychological treatment for BPD with sufficient data to pool into a meta-analysis (Stoffers et al. 2012). Results demonstrated moderate to large statistically significant effects for DBT over treatment as usual in reductions in suicidal and self-harm behaviours (SMD −0.54, 95 % CI −0.92 to −0.16); improvements in mental health (SMD 0.65, 95 % CI 0.07–1.24) and decreases in anger (SMD −0.83, 95 % CI −1.43 to −0.22). The National Institute for Health and Care Excellence (NICE) Guidelines for the treatment and management of BPD recommend DBT particularly where reduction in self-harm is a clinical priority. An earlier review reported that DBT had the potential for cost-effectiveness (Brazier et al. 2006).

As a direct consequence of its success in treating suicidal and self-harm behaviours with adults diagnosed with BPD, DBT was adapted for the treatment of adolescents (DBT-A) presenting with suicidal and self-harm behaviours who were also demonstrating features of a developing BPD (Miller et al. 2007). A recent RCT of the adapted form of DBT conducted in Norway replicated the findings of earlier studies in adult populations. Mehlum et al. (2014) randomly allocated 77 adolescents presenting with suicidal and self-harming behaviour with at least two other BPD characteristics to either DBT-A or Enhanced Treatment as Usual (ETAU). After 16 weeks of treatment DBT-A was significantly superior to ETAU in terms of decreases in self-harm behaviour and depression. Several non-randomised studies conducted in the UK and elsewhere also indicate that DBT maybe a promising intervention with adolescents presenting with self-harm behaviour in the context of BPD (Fleischhaker et al. 2011; James et al. 2008, 2011; Katz et al. 2004).

Adults diagnosed with BPD have severe impairments in Health Related Quality of Life (HRQoL) (van Asselt et al. 2009; Soeteman et al. 2008; Perseius et al. 2006). In addition to the significant personal burden of a diagnosis of BPD (Heard 2000), clients with BPD are typically “treatment seeking” with associated high utilisation of health services (Bender et al. 2001; Benjamin 1993; Coid 2003; Lieb et al. 2004), leading researchers and policy makers alike to highlight the importance of investigating and implementing clinically and cost-effective treatments for this population (Esposito-Smythers et al. 2011; Soeteman et al. 2008). Establishing cost-effectiveness (specifically cost-utility analysis) requires generic preference based measures that can be used to calculate the cost per additional Quality-adjusted life year (QALY). QALYs are a generic measure of disease burden that includes both the quality and length of life and allow for comparison across conditions to help inform decision makers where best to invest scarce health care resources. NICE (NCCMH 2009) recommend the EuroQol 5 Dimensions (EQ-5D™) as the most appropriate measure for health economic evaluations of new technologies. This standardised and validated self-report measure describes an individual’s current health status and can be used to identify changes occurring over time. The construct validity and test–retest reliability of the EQ-5D™ have also been supported (van Asselt et al. 1994).

As the measure is generic rather than condition-specific, the EQ-5D™ provides a common denominator for different evaluations allowing comparison of new technologies with each other. Such generic measures assess broad levels of functioning in contrast to symptomatic measures that may address a single clinical outcome e.g. self-harm or depression. Whilst individual symptom measures are an important measure of clinical outcome, clinical guidance recommends considering broader measures of functioning and quality of life rather than simply symptomatic improvement (NCCMH 2009). Use of generic measures maybe of particular importance in the case of BPD where clients’ problems impinge on a wide range of health domains.

Using the EQ-5D has an additional advantage above addressing functional outcomes more broadly; measures that enable the calculation of QALYs across a number of services will provide data about cost-effectiveness of DBT as delivered in routine clinical practice, and, subject to sufficient variation across programmes, allow for the development of a national benchmarking system. Transferring evidence-based treatments established as efficacious in randomised-controlled clinical trials to routine clinical practice is fraught with difficulty (Damschroder et al. 2009). Routinely measuring outcomes in clinical settings and benchmarking them against published efficacy data provides one method of assessing whether clients are benefiting from the treatments they receive. Once programmes that do not deliver good clinical outcomes can be identified, organisational interventions to address poor outcomes can be developed and implemented.

The primary aim of the pilot study was to evaluate the HRQoL outcomes of adolescents receiving DBT in routine clinical practice. Our secondary aim, given the absence of studies reporting on HRQoL in adolescents, was to compare the findings with published RCT data on adults with BPD and adolescents with other mental health conditions. Finally, we wished to assess the potential of the EQ-5D™ as a benchmarking measure.

## Methods

### Procedure

All DBT programmes (N = 9) with a subscription to the DBT pseudonymised outcome benchmarking website at www.dbt.uk.net were invited to participate in the study. The pseudonoymised outcome website was developed to assist teams to collect routine outcome data based on the potential value of such data for implementation of DBT programmes (Swales et al. 2012). To reduce the administrative burden on participating programmes and hopefully maximise the success of data collection, the amount of data required was kept to an absolute minimum using a single outcome measure, the EQ-5D™. Consistent with keeping the demands on busy programmes low, only pre- and post-treatment data were required for entry on the website. Programmes were asked to assess clients on the EQ-5D™ at admission to the programme and on discharge, regardless of whether discharge was planned or unplanned. Assessing all entrants to the programme, regardless of whether they complete treatment or not, provides a more conservative test of the effectiveness of a treatment programme. Only including data from treatment completers may overestimate the effectiveness of treatment in routine practice. Pseudonyms were selected according to the gender of the client so number of male and female clients in the sample as a whole is known; otherwise no demographic data is available at the individual client level.

Seven of the subscribed nine teams were working with adolescents. A census of DBT clients in treatment on a specified date (1 August 2011) established that only four of these programmes were using the website to routinely collect data with sufficient accuracy for benchmarking purposes, namely that the website was reporting the correct number of clients in treatment that day. Length of time programmes had used the website for routine data collection varied between 7 (January 2011) and 16 months (April 2009). These four programmes consented for their data to be downloaded from the system on the predefined census date and for their programme data to be included in the multi-site data analysis. All participating programmes had broadly similar admission criteria (ages 14–18; five or more BPD criteria of which one must be the recent occurrence of self-harm behaviour).

All programmes had permission from their information governance officers to upload pseudonymised routine outcomes to the website. Ethical Approval was sought and received from the Ethics Committee of the School of Psychology, Bangor University. The Local NHS Research Ethics Committee was asked for an opinion on whether the study required NHS approval and considered that the study qualified as service evaluation. No patient identified information was submitted to or held by the outcome benchmarking website.

### Measures

#### EQ-5D™

 The EQ-5D™ is a generic outcome measure that asks participants to rate their current general health status on 5 dimensions: mobility, self-care, usual activities, pain or discomfort and anxiety and depression. The participant rates each of these dimensions at one of three different levels (no problems, some problems or extreme problems). The scoring system then classifies the individual into one of 243 possible health states. Each health state provides a summary of the participants rating of their health status on each dimension. For example, the health state ‘11111’ represents perfect health on all 5 dimensions—a state that 56 % of the UK population and 70 % of the Spanish population will report on any one day (Feng et al. 2015; Gaminde and Roset 2001). In contrast, a health status of ‘33333’ indicates the worst possible health status with extreme problems on all 5 dimensions. These scores can be transformed into a utility score that ranges between −0.59 and 1 (with death anchored at 0 and 1 representing perfect health) by applying societal weights to each level that are based on the values of these health states in adult general population samples derived from a choice based method such as time trade-off (TTO) (Szende et al. 2007). Currently there are no weights derived from valuations of health states by children and adolescents. Negative scores indicating health states judged as ‘worse than death’ are possible. The utility scores can be used to calculate QALYs. The sole adolescent study utilising the EQ-5D™ provided tentative support for its use in this client group (Byford 2013).

### Data collection and entry

Each DBT team had its own protocol for administering and entering the data onto the website. In most cases young people scored the questionnaires themselves. On some occasions clinicians completed the questionnaires on their behalf. Proxy completion of the EQ-5D™ is a recognised method of data collection. In a recent study of the EQ-5D™ with children and adolescents from a community study, where parents acted as proxies, high levels of agreement were found between the self-report and proxy versions of the EQ-5D-Y (Gusi et al. 2014). Each DBT team uploaded EQ-5D scores for all patients treated in their DBT programme at admission and discharge. An audit on a random selection of inputs from each team was conducted to ensure that the data had been accurately entered into the website. To conduct the audit one of the research team (LB) telephoned the DBT team and asked them to provide the EQ-5D™ scores stored on paper in the clinical notes for each client for a set of randomly chosen patients and time points. These scores were checked for accuracy with the data already entered into the website. No inconsistencies between the data entered onto the system and the original paper copies of the data were detected during this audit.

### Data analysis strategy

Utility scores at or below zero might be anticipated in clinical samples for which achieving a ‘life worth living’ (Linehan 1993) remains a daily struggle. In a sample of adolescents in treatment for suicidal and self-harm behaviours a measure that captures health states considered ‘worse than death’ displays considerable face and criterion validity; these scores were retained. Difference scores were calculated by subtracting EQ-5D™ utility scores on discharge from those at admission.

In an endeavour to establish whether the change in EQ-5D™ scores represented a robust change at the individual level, the Reliable Change Index for the EQ-5D was calculated using the Jacobson method (Jacobson and Truax 1991). This formula takes into account the reliability of the measure and variance in measurement to generate a change score in excess of which we can essentially be 95 % certain that the change in score is a real (hence, reliable) change over time. Ideally, in order to exclude sources of systematic error in this calculation, an estimate of test-retest reliability derived from a clinical sample whose clinical characteristics have not changed over a period of time would be preferred. Since clinical samples are, by definition, in treatment and therefore on a change trajectory and the EQ-5D™ is not yet used widely in mental health settings, such estimates are hard to obtain. Hurst et al. (1997, Table IX), using an intraclass correlation coefficient (ICC) rather than Pearson correlation because of concerns about over-estimating reliability, reported a reliability coefficient for the EQ-5D™ of 0.78 (0.6–0.96) over 2 weeks in a clinical sample of 31 rheumatoid arthritis sufferers where there was no change in rheumatoid arthritis. More recently Sonntag et al. (2013, Table 4) have tabulated ICCs for n = 106 social phobics at an interval of 6 months and n = 60 at 12 months, anchored by no change on the Liebowitz Social Anxiety Scale which are all also 0.78. In view of the replication of this estimate and albeit limited clinical similarities in the study populations, this value was used in the calculation of the Reliable Change Index in the DBT programmes in this study.

## Results

### Description of the sample

On the census date, 76 sets of client data were downloaded from four programmes of which 66 had female pseudonyms and 10 male. Of these 76 clients, 43 (38 female and 5 male) had been discharged from their respective programme and 33 were still in treatment. Admission EQ-5D™ utility scores for the whole sample (n = 76) were 0.236 (SD 0.32, range −0.594 to 0.848). The admission EQ-5D scores of the 43 adolescents who had completed treatment prior to the census date were not significantly different from the 33 young people who were still in treatment. [Mean admission utility score still in treatment (n = 33) = 0.244, mean admission utility score discharged (n = 43) = 0.230, t(75) = 0.19, p = 0.85.] Average length of stay of adolescents consecutively discharged from programmes (n = 43) was 177 days (SD 116, range 23–462).

### Comparison of admission and discharge HRQoL scores

Admission and discharge scores for the 43 consecutive discharges in the dataset were compared. Mean admission utility scores were 0.230 (SD 0.345, range −0.590 to 0.883) and mean discharge utility scores were 0.554 (SD 0.376, range −0.008 to 1.000). Fourteen clients reported health states as worse than death at admission and nine at discharge (Table 1). These data were not normally distributed and were tested for significance using the Wilcoxon signed-rank test. Utility scores between admission and discharge were significantly different (z = −4.26, p < 0.001).

**Table 1:** Comparison of EQ-5D scores across studies

Sample	EQ-5D at t1 Mean (95 % confidence intervals)	EQ-5D at t2 Mean (95 % confidence intervals)	t2 − t1 (mean length of treatment)	Average difference	Effect size d
Present study: n = 43 routine admissions to DBT programmes	0.23 (0.12–0.34) EQ-5D < 0 33 %^a^	0.55 (0.44–0.67) EQ-5D < 0 21 %^a^	10 months	0.32 (0.20–0.44) p < 0.001	1.00
RCTs in adults					
Nadort et al. (2009) n = 62 SFT versus modified SFT for adult BPD	0.44 (0.31–0.56)	0.56 (0.47–0.65)	18 months	0.12 (0.02–0.22) p = 0.02	0.39^b^
van Asselt et al. (2008) n = 48 transference versus SFT for adult BPD	0.50 (0.41–0.59)	0.69 (0.61–0.77)	3 years	0.20 (0.09–0.29) p < 0.001	0.64
RCTs in CAMHS					
Byford (2013) n = 199 CBT/SSRI versus SSRI for adolescent depression	0.50 (0.46–0.54) EQ-5D < 0 5.5 %	0.76 (0.72–0.80) EQ-5D < 0 1.5 %	28 weeks	0.26 (not reported)	0.90

^a^Includes 9 clients (21 %) admitted in state 11233 whose utility remained unchanged at −0.008

^b^Relative to baseline variance which is the effect size commonly used in power calculations, Nadort et al. (2009) calculate effect sizes relative to pooled variance (pre/post) and report a lower estimate of 0.35

Variability between DBT programmes in baseline health status on admission (p < 0.001) and their ability to generate changes in EQ-5D scores between admission and discharge (p < 0.0001) was apparent in the dataset. This was indicative of clustering in the data that would exaggerate the significance levels in analyses if not accounted for. An intra-programme correlation coefficient was calculated from the difference scores (treating DBT programme as a random effect) and used to inflate the width of the estimated confidence interval for mean differences to account for between-programme variability in outcomes.

The high-level of clustering, indicated by an ICC of 0.71, increased the variance of the average difference score by a factor of approximately 12 according to the exact method of calculation for unequal cluster sizes given by Donner, Birkett and Buck (Donner et al. 1981). The average difference in EQ-5D™ utility scores of 0.32 between admission and discharge failed to attain statistical significance once this adjustment was made (t = 1.56, p = 0.13).

The Reliable Change Index calculation indicated that EQ-5D™ difference scores of at least 0.45 could be considered reliable in the present study. Seventeen clients in the sample of 43 (40 %) experienced reliable change between admission and discharge. The ability of clients to achieve change on the EQ-5D™ was constrained by an obvious ceiling effect in that over 25 % in the DBT sample had a utility score >0.55 on admission and so could not achieve a change of >0.45.

### Comparison of HRQoL scores with other published data sets

Ishak et al. (2013) identified only 10 studies of psychotherapeutic interventions for BPD incorporating the use of HRQoL measures, of which only three report the EQ-5D™ as an outcome measure (McMain et al. (2009) analyse the Euroqol VAS thermometer but do not report the EQ-5D utility scores) and, of these remaining three, only two represent unique datasets (van Asselt et al. (2009) re-analyse the Giesen-Bloo et al. (2006) dataset). Both are studies in adult BPD. There is currently limited, but promising evidence to support the use of the EQ-5D instrument in CAMHS settings as a HRQoL measure (Byford 2013).

Baseline and discharge EQ-5D™ scores for this clinical sample of admissions are presented in Table 1, alongside pre-and post-intervention EQ-5D™ scores from the few available RCTs in mental health for comparison purposes. As the present study is a small-uncontrolled study and primarily of clinical interest in relation to benchmarking outcomes across DBT programmes, effect sizes using Cohen’s d are also reported. The EQ-5D™ scores at treatment commencement are much lower in this study than in the studies of BPD in adult populations (Nadort et al. (2009), t(103) = 2.15, p < 0.05; van Asselt et al. (2008) t(89) = 2.70, p < 0.005), both of which were of Schema-Focussed therapy. The adolescents in this pilot study end treatment at an EQ-5D™ score average commensurate with the starting EQ-5D™ scores of adolescents in the RCT for depression (Byford (2013)t(241) = 0.88, p > 0.02, ns). The effect size for this pilot study is higher than the two adult studies (Nadort et al. 2009; van Asselt et al. 2008) but a similar size to the adolescent depression study (Byford 2013).

## Discussion

This study is the first to report on HRQoL of adolescents with BPD-type presentations in routine clinical practice. Participants in the DBT programmes in this study had significantly impaired health related quality of life (HRQoL) on admission, scoring significantly lower than data available from a published RCT using the EQ-5D with adult BPD participants (van Asselt et al. 2009) and from a study reporting on the treatment of adolescents with depression (Byford 2013). These data potentially support the often-expressed view of clinicians that clients in clinical services are more severe than those participating in clinical trials. Indeed, this view has been highlighted as a barrier to the implementation of evidence-based practice (Shafran et al. 2009).

Alternatively the experience of BPD in adolescence compared to adulthood may have a particularly significant effect on HRQoL. Typically adolescents presenting with developing BPD in adolescence have experienced high levels of adversity in a context of genetic vulnerability over many years (Sharp and Fonagy 2015). In addition to significant mental health difficulties with high rates of comorbidity (Chanen et al. 2007; Ha et al. 2014), they encounter major problems at school and with familial relationships and friends (Winograd et al. 2008; Zanarini et al. 2006; Crawford et al. 2008) that persist often into adulthood even when some of the more impulsive behaviours may have subsided. In clinical practice the broad ranging impact on almost all aspects of development is striking. Poor HRQoL in this context is perhaps unsurprising.

Despite the severity of impairment demonstrated in the patients treated in the DBT programmes, their HRQoL improved between admission and discharge with 40 % of the sample achieving Reliable Clinical Change. This finding is particularly interesting when considering the fact that patients were discharged for multiple reasons—including both planned (e.g. they completed the DBT programme and no longer needed DBT) and unplanned discharges (e.g. the patient chose to stop attending treatment). This study reported on the changes between admission and discharge for all patients who had received DBT regardless of whether they completed the treatment programme or not. This finding also runs counter to the views clinicians express (Shafran et al. 2009) that increased clinical severity might prevent significant and reliable clinical change.

Despite the change in HRQoL scores for patients in the DBT programmes, discharge scores remained considerably lower than the general population norm and comparable to the scores of adolescents *commencing* treatment in an RCT for depression (Byford 2013). These findings underscore the view, reported in the literature, that individuals with BPD have significant impairment of HRQoL (van Asselt et al. 2009; Soeteman et al. 2008; Perseius et al. 2006) even following what is an effective treatment. The results suggest that further research should be directed towards further enhancing clinical outcomes. Typically, adaptations of DBT for adolescents have shortened the programme duration from 1 year (typical in adult services) to 16 weeks (Mehlum et al. 2014; Brazier et al. 2006). The final HRQoL outcomes in this study would argue against this given the low level of functioning of adolescents at treatment end and that adolescents in this study had a longer average treatment length than that described in the literature (Mehlum et al. 2014; Miller et al. 2007). In the shorter forms of DBT-A adolescents entering the programme typically have fewer BPD symptoms (typically 2–3) whereas in the clinical programmes studied here inclusion criteria to the programme required 5 or more BPD criteria which may account for the longer treatment duration. These results suggest that for adolescents at this level of severity longer treatment durations may be necessary.

Not all of the programmes were equally successful in producing good HRQoL outcomes. When this variability was accounted for outcomes were not significantly different at treatment end. The level of clustering in the dataset (ICC of 0.71) is unusually high, beyond the range commonly encountered in naturally occurring biological and disease-related phenomena. Such variability is perhaps not uncommon in pilot data, especially with a team-based treatment delivered within highly variable organisational contexts. Whether the high levels of clustering were driven by differences in programme fidelity or therapist competence or are simply the artefact of small numbers of participants with a condition with highly variable outcomes is unknown. Future studies with more programmes with larger numbers of teams and treated clients will be necessary to tease out this finding. Paradoxically, the differential success of the DBT programmes in producing HRQoL outcomes, with some programmes performing better than others, augurs well for the intended use of the website for future benchmarking between programmes.

Whilst these findings in this pilot study are of interest, aspects of the data collection indicate that they should be interpreted with a significant degree of caution. Firstly, only four out of seven CAMHS teams with a subscription to the website were successfully using the system to collect routine data. The teams that were collecting data may have been especially motivated and thus potentially have been more likely to produce improved outcomes. Resolving problems in routinely collecting outcomes using the system would be essential for future meaningful use of the system to collect national outcome data or to benchmark programmes systematically. Secondly, these data were collected under routine clinical practice conditions and so the research team did not control data collection and entry. Each individual DBT programme operated their own data administration, collection and entry procedures for their own clinical purposes and this may have led to different protocols for data collection. In some teams the proportion of EQ-5Ds completed by clinicians as a proxy, may have been higher and in some cases clinicians recorded the measure retrospectively, particularly in circumstances where patients left the programme prematurely. Both of these practices may have resulted in biases in the data. Secondly, the absence of any additional data on either the participants or other outcomes limits generalizability. Thirdly, the absence of a control group means that change in the sample cannot be attributed to the treatment that they received. The absence of a control group and the high-level of clustering means that these data cannot make any definitive statement about whether DBT is effective in adolescents with BPD treated in routine clinical practice.

In conclusion, although further research is necessary to unpick the findings of this pilot study, the DBT outcome monitoring website has demonstrated its potential to collect data in routine practice and in real-time and thus may be a promising tool to benchmark what is gained and lost following the implementation of DBT in clinical practice settings.

## References

[CR1] Bender DS, Dolan RT, Skodol AE, Sanislow CA, Dyck IR, McGlashan TH, Shea MT, Zanarini MC, Oldham JM, Gunderson JG (2001). Treatment utilization by patients with personality disorders. Am J Psychiatry.

[CR2] Benjamin LS (1993). Interpersonal diagnosis and treatment of personality disorders.

[CR3] Brazier J, Tumur L, Holmes M, Ferriter M, Parry G, Dent-Brown K, Paisley S (2006) Psychological therapies including dialectical therapy for borderline personality disorder: a systematic review and preliminary economic evaluation. Health Technol Assess 10(35) 10.3310/hta10350 16959171

[CR4] Byford S (2013). The validity and responsiveness of the EQ-5D measure of health-related quality of life in an adolescent population with persistent major depress. J Ment Health.

[CR5] Chanen AM, Jovev M, Jackson HJ (2007). Adaptive functioning and psychiatric symptoms in adolescents with borderline personality disorder. J Clin Psychiatry.

[CR6] Clarkin JF, Levy KN, Lenzenweger MF, Kernberg OF (2007). Evaluating three treatments for borderline personality disorder: a multiwave study. Am J Psychiatry.

[CR7] Cohen P, Crawford TN, Johnson JG, Kasen S (2005). The children in the community study of developmental course of personality disorder. J Pers Disord.

[CR8] Coid JW (2003). The co-morbidity of personality disorder and lifetime clinical syndromes in dangerous offenders. J Forens Psychiatry Psychol.

[CR9] Crawford TN, Cohen P, First MB, Skodol AE, Johnson JG, Kasen S (2008). Comorbid Axis I and Axis II disorders in early adolescence: outcomes 20 years later. Arch Gen Psychiatry.

[CR10] Damschroder LJ, Aron DC, Keith RE, Kirsh SR, Alexander JA, Lowery JC (2009). Fostering implementation of health services research findings into clinical practice: a consolidated research framework for advancing implementation. Implement Sci.

[CR11] Donner A, Birkett N, Buck C (1981). Randomization by cluster: sample size requirements and analysis. Am J Epidemiol.

[CR12] Esposito-Smythers C, Spirito A, Kahler C, Hunt J, Monti P (2011). Treatment of co-occurring substance abuse and suicidality among adolescents: a randomised trial. J Consult Clin Psychol.

[CR13] Feng Y, Devlin N, Herdman M (2015). Assessing the health of the general population in England: how do the three-level and five-level versions of EQ-5D compare?. Health Qual Life Outcomes.

[CR14] Fleischhaker C, Bohme R, Sixt B, Bruck C, Schneider C, Schulz E (2011). Dialectical behavioral therapy for adolescents (DBT-A): a clinical trial for patients with suicidal and self-injurious behaviour and borderline symptoms with a one-year follow-up. Child Adolesc Psychiatry Ment Health.

[CR15] Gaminde I, Roset M (2001) Quality adjusted life expectancy. In: Cabasés J, Gaminde I (eds) 17th plenary meeting of the EuroQol Group. Discussion papers. Universidad Pública de Navarra, pp 173–183

[CR16] Giesen-Bloo J, van Dyck R, Spinhoven P, van Tilburg W, Dirksen C, van Asselt T, Kremers I, Nadort M, Arntz A (2006). Outpatient psychotherapy for borderline personality disorder randomized trial of schema-focused therapy vs transference-focused psychotherapy. Arch Gen Psychiatry.

[CR17] Gusi N, Perez-Sousa MA, Gozalo-Delgado M, Olivares PR (2014). Validity and reliability of the spanish EQ-5D-Y proxy version. An Pediatr.

[CR18] Ha C, Balderas JC, Zanarini MC, Oldham J, Sharp C (2014). Psychiatric comorbidity in hospitalised adolescents with borderline personality disorder. J Clin Psychiatry.

[CR19] Heard HL (2000) Cost-effectiveness of dialectical behavior therapy in the treatment of borderline personality disorder. Doctoral dissertation, University of Washington

[CR20] Hurst NP, Kind P, Ruta D, Hunter M, Stubbings A (1997). Measuring health-related quality of life in rheumatoid arthritis: validity, responsiveness and reliability of EuroQol (Eq-5d). Br J Rheumatol.

[CR21] Ishak WW, Elbau I, Ismail A, Delaloye S, Ha K, Bolotaulo NI, Nashawati R, Cassmassi B, Wang C (2013). Quality of life in borderline personality disorder. Harv Rev Psychiatry.

[CR22] Jacobson NS, Truax P (1991). Clinical significance: a statistical approach to defining meaningful change in psychotherapy research. J Consult Clin Psychol.

[CR23] James AC, Taylor A, Winmill L, Alfoadari K (2008). A preliminary community study of dialectical behaviour therapy (DBT) with adolescent females demonstrating persistent, deliberate self-harm (DSH). Child Adolesc Ment Health.

[CR24] James AC, Winmill L, Anderson C, Alfoadari K (2011). A preliminary study of an extension of a community dialectic behaviour therapy (DBT) programme to adolescents in the looked after care system. Child Adolesc Ment Health.

[CR25] Katz LY, Cox BJ, Gunasekara S, Miller AL (2004). Feasibility of dialectical behaviour therapy for suicidal adolescent inpatients. J Am Acad Child Adolesc Psychiatry.

[CR26] Koons CR, Robins CJ, Tweed JL, Lynch TR, Gonzalez AM, Morse JQ, Bishop GK, Butterfield M, Bastian LA (2001). Efficacy of dialectical behavior therapy in women veterans with borderline personality disorder. Behav Ther.

[CR27] Lieb K, Zanarini M, Linehan MM, Bohus M (2004). Seminar section: borderline personality disorder. Lancet.

[CR28] Linehan MM (1993). Cognitive-behavioural treatment of borderline personality disorder.

[CR29] Linehan MM, Armstrong HE, Suarez A, Allman D, Heard HL (1991). Cognitive-behavioral treatment of chronically parasuicidal borderline patients. Arch Gen Psychiatry.

[CR30] Linehan MM, Schmidt H, Dimeff LA, Craft JC, Kanter J, Comtois KA (1999). Dialectical behavior therapy for patients with borderline personality disorder and drug dependence. Am J Addict.

[CR31] Linehan MM, Dimeff LA, Reynolds SK, Comtois KA, Shaw-Welch S, Heagerty P, Kivlahan DR (2002). Dialectical behavior therapy versus comprehensive validation plus 12- step for the treatment of opioid dependent women meeting criteria for borderline personality disorder. Drug Alcohol Depend.

[CR32] Linehan MM, Comtois KA, Murray AM, Brown MZ, Gallop RJ, Heard HH, Korslund KE, Tutek DA, Rynolds SK, Lindenboim N (2006). Two-year randomized controlled trial and follow-up of dialectical behavior therapy vs therapy by experts for suicidal behaviors and borderline personality disorder. Arch Gen Psychiatry.

[CR33] McMain SF, Links PS, Gnam WH, Guimond T, Cardish RJ, Korman L, Streiner DL (2009). A randomized trial of dialectical behavior therapy versus general psychiatric management for borderline personality disorder. Am J Psychiatry.

[CR34] Mehlum L, Tørmoen AJ, Ramberg M, Haga E, Diep LM, Laberg S, Grøholt B (2014). Dialectical behavior therapy for adolescents with repeated suicidal and self-harming behavior: a randomized trial. J Am Acad Child Adolesc Psychiatry.

[CR35] Miller AL, Rathus JH (2002). Dialectical behavior therapy adapted for suicidal adolescents. Suicide Life Threat Behav.

[CR36] Miller AL, Rathus JH, Linehan MM (2007). Dialectical behavior therapy with suicidal adolescents.

[CR37] Miller AL, Muhlenkamp JJ, Jacobson CM (2008). Fact or fiction: diagnosing borderline personality disorder in adolescents. Clin Psychol Rev.

[CR38] Nadort M, Arntz A, Smith JH, Giesen-Bloo J, Eikelenboom M, Spinhoven P, van Asselt T, Wensing M, van Dyck R (2009). Implementation of an outpatient schema therapy for borderline personality disorder with versus without crisis support by the therapist outside office hours: a randomized trial. Behav Res Ther.

[CR39] NCCMH (2009). Borderline personality disorder: treatment and management.

[CR40] Ougrin D, Tranah T, Stahl D, Moran P, Asarnow JR (2015). Therapeutic interventions for suicide attempts and self-harm in adolescents: systematic review and meta-analysis. J Am Acad Child Adolesc Psychiatry.

[CR41] Perseius KI, Andersson E, Asberg M, Samuelsson M (2006). Health-related quality of life in women patients with borderline personality disorder. Scand J Caring Sci.

[CR42] Roussow T, Fonagy P (2012). Mentalisation-based treatment for self-harm in adolescents: a randomized controlled trial. J Am Acad Child Adolesc Psychiatry.

[CR43] Shafran R, Clark DM, Fairburn CG, Arntz A, Barlow DH, Ehlers A (2009). Mind the gap: improving the dissemination of CBT. Behav Res Ther.

[CR44] Sharp H, Fonagy P (2015). Practitioner review: borderline personality disorder in adolescence—recent conceptualization, intervention, and implications for clinical practice. J Child Psychol Psychiatry.

[CR45] Soeteman DI, Verheul R, Busschbach JJV (2008). The burden of disease in personality disorders: diagnosis-specific quality of life. J Pers Disord.

[CR46] Sonntag M, Konnopka A, Leichsenring F, Salzer S, Beutel ME, Herpertz S, Hiller W, Hoyer J, Joraschky P, Nolting B, Pöhlmann K, Stangier U, Strauss B, Willutzki U, Wiltink J, Leibing E, König H (2013). Reliability, validity and responsiveness of the EQ-5D in assessing and valuing health status in patients with social phobia. Health Qual Life Outcomes.

[CR47] Stoffers JM, Völm BA, Rücker G, Timmer A, Huband N, Lieb K (2012) Psychological therapies for people with borderline personality disorder. Cochrane Libr 8. doi:10.1002/14651858.CD005652.pub2 10.1002/14651858.CD005652.pub2 PMC6481907 22895952

[CR48] Swales MA, Taylor B, Hibbs RAB (2012). Implementing Dialectical Behaviour Therapy: programme survival in routine healthcare settings. J Ment Health.

[CR49] Szende A, Oppe M, Devlin N (2007). EQ-5D value sets: inventory, comparative review and user guide.

[CR50] van Asselt AD, Essink-Bot M, Krabbe PFM, Bonsel GJ (1994). Test-retest reliability of health state valuations collected with the EuroQol questionnaire. Soc Sci Med.

[CR51] van Asselt AD, Dirksen CD, Arntz A, Giesen-Bloo JH, van Dyke R, Spinhoven P, van Tilburg W, Kremers IP, Nadort M, Severens JL (2008). Out-patient psychotherapy for borderline personality disorder: cost-effectiveness of schema- focused therapy v. transference-focused psychotherapy. Br J Psychiatry.

[CR52] van Asselt AD, Dirksen CD, Arntz A, Giesen-Bloo JH, Severens JL (2009). The EQ-5D: a useful quality of life measure in borderline personality disorder?. Eur Psychiatry J Assoc Eur Psychiatr.

[CR53] Verheul R, Van Den Bosch LMC, Koeter MWJ, De Ridder MAJ, Stijnen T, Van Den Brink W (2003). Dialectical behaviour therapy for women with borderline personality disorder: 12-month, randomised clinical trial in The Netherlands. Br J Psychiatry.

[CR54] Winograd G, Cohen P, Chen H (2008). Adolescent borderline symptoms in the community: prognosis for functioning over 20 years. J Child Psychol Psychiatry.

[CR55] Zanarini MC, Frankenberg FR, Hennen J, Reich DB, Silk KR (2006). Prediction of the 10 year course of borderline personality disorder. Am J Psychiatry.

